# Acute Toxicity, Respiratory Reaction, and Sensitivity of Three Cyprinid Fish Species Caused by Exposure to Four Heavy Metals

**DOI:** 10.1371/journal.pone.0065282

**Published:** 2013-06-03

**Authors:** Hongjun Wang, Youguang Liang, Sixin Li, Jianbo Chang

**Affiliations:** Key Laboratory of Ecological Impacts of Hydraulic-Projects and Restoration of Aquatic Ecosystem of Ministry of Water Resources, Institute of Hydroecology, Ministry of Water Resources and Chinese Academy of Sciences, Wuhan, P.R. China; Pacific Northwest National Laboratory, United States of America

## Abstract

Using 3 cyprinid fish species zebra fish, rare minnow, and juvenile grass carp, we conducted assays of lethal reaction and ventilatory response to analyze sensitivity of the fish to 4 heavy metals. Our results showed that the 96 h LC_50_ of Hg^2+^ to zebra fish, juvenile grass carp, and rare minnow were 0.14 mg L^−1^, 0.23 mg L^−1^, and 0.10 mg L^−1^, respectively; of Cu^2+^0.17 mg L^−1^, 0.09 mg L^−1^, and 0.12 mg L^−1^ respectively; of Cd^2+^6.5 mg L^−1^, 18.47 mg L^−1^, 5.36 mg L^−1^, respectively; and of Zn^2+^44.48 mg L^−1^, 31.37 mg L^−1^, and 12.74 mg L^−1^, respectively. Under a 1-h exposure, the ventilatory response to the different heavy metals varied. Ventilatory frequency (Vf) and amplitude (Va) increased in zebra fish, juvenile grass carp, and rare minnows exposed to Hg^2+^ and Cu^2+^ (P<0.05), and the Vf and Va of the 3 species rose initially and then declined when exposed to Cd^2+^. Zn^2+^ had markedly different toxic effects than the other heavy metals, whose Vf and Va gradually decreased with increasing exposure concentration (P<0.05). The rare minnow was the most highly susceptible of the 3 fish species to the heavy metals, with threshold effect concentrations (TEC) of 0.019 mg L^−1^, 0.046 mg L^−1^, 2.142 mg L^−1^, and 0.633 mg L^−1^ for Hg^2+^, Cu^2+^, Cd^2+^, and Zn^2+^, respectively. Therefore, it is feasible to use ventilatory parameters as a biomarker for evaluating the pollution toxicity of metals and to recognize early warning signs by using rare minnows as a sensor.

## Introduction

With the development of industry and agriculture, numerous heavy metal pollutants have been released into water bodies by various means, resulting in serious water pollution. In polluted waters, exposure of fish to heavy metals leads to interactions between these chemicals and biological systems and causes biochemical disturbances[1]–[2]. Mercury (Hg), copper (Cu), cadmium (Cd), and zinc (Zn) are the 4 most common heavy metals. Hg bioaccumulates in organisms, especially in carnivorous fish at high trophic levels of the food chain, and its concentration can become tens of thousands-fold greater than that in water. In the early 1960s, Songhua River (China) was seriously polluted, and it became a water body that typified Hg pollution from discharge of industrial wastewater [3]. Cd is a nonessential heavy metal of high environmental concern due to its toxicity, common usage, industrial production, and emissions from fossil fuel combustion. Cd is on some governments'priority-substance lists (e.g., Canadian Environmental Protection Act, 1994), and in environments impacted by man, concentrations may reach values of micrograms per liter or higher [4]. Cu is an essential element, required by all living organisms for several physiological functions and biochemical reactions [5]. Zn is also an essential trace element, but excess of both Cu and Zn is poisonous to organisms.

Using fish as biological indicators is advantageous because changes in their behaviors (e.g., avoidance responses, swimming patterns, and breathing) in response to environmental changes can be measured directly. Indeed, behavior has been used as an integral parameter of physiological activity and as a robust biological warning indicator of the quality of water supplies and effluents[6]–[7]. Fish ventilatory parameters that are known to be sensitive to toxins include ventilatory rate (opercular movement), depth or amplitude of ventilation, and coughing or gill purge rate [8].

Over the past 40 years, an early warning system was successfully developed by using fish as bioindicators and the ventilatory parameters of fish were used as biomarkers for online monitoring of water pollution[9]–[10]. Numerous studies found that this warning system was sensitive to the resource water and to emission of pollutants [11]. The system was used in Europe where it has been successfully applied in several rivers since 1990[12]–[13], and has been extensively applied in South Africa [14]. In response to the September 11, 2001 terrorist attacks in the United States (US) and a subsequent anthrax bacteria event, the US began to pay attention to the research and application of biological early warning systems [6]. However, such studies are still lacking in China.

Related research on fish behavior toxicology indicated that ventilatory behavior was sensitive to heavy metals. Many researchers primarily used bluegill sunfish[8]–[9] and rainbow trout [11] to conduct toxicological experiments. A review summarizing the methodology for measurement and interpretation of fish ventilatory patterns as early warning signals of water quality deterioration and incipient toxicity was available [15]. In the work presented here, we used standard experimental fish – zebra fish, a rare local minnow, and grass carp – for a ventilatory toxicology experiment examining 4 heavy metals. We analyzed the sensitivity of 4 heavy metals in experimental fish and considered how the respiratory parameters of local rare minnow could provide basic data for monitoring and giving early warning of heavy metal pollution in water bodies.

## Materials and Methods

### Experimental Animals

The 3 cyprinid fish species used in the present study included 2 native, local Chinese species: juvenile grass carp (Ctenopharyngodon idellus) and rare minnow (Gobiocypris rarus), and one introduced, standard international experimental fish: zebra fish (Brachydanio rerio). The mean lengths and weight (± standard deviation) of zebra fish, juvenile grass carp, and rare minnow were 2.3±0.3 cm and 0.22±0.05 g, 4.1±0.2 cm and 1.84±0.33 g, and 2.6±0.7 cm and 0.25±0.06 g, respectively. G. rarus and B. rerio were obtained from Institute of Hydrobiology, Chinese Academy of Sciences, China, and C. idellus was obtained from a fish hatchery in Hubei Province, China. The death rate of experimental fish, which were raised for 14 days and fasted for 24 hours before the experiment, was less than 2%. All procedures and animal handling were in accordance with the guideline approved by Chinese Association For Laboratory Animal Sciences. The study was approved by the animal ethics committee of the Institute of Hydroecology, Ministry of Water Resources and Chinese Academy of Sciences (protocol number: IHE20110525).

After the experiment, fish that had gone through the heavy metal treatment and were less active or stressed were euthanized using overdose of Benzocaine. The surviving fish that did not have symptoms of poison were put in clean fresh water and closely monitored. No fish died during the ventilatory monitoring experiments.

### Dilution Water and Heavy Metals

The tap water used in the experiment was aerated, dechlorinated, and charged with oxygen for more than 48 h. Dissolved oxygen, pH, conductivity, total hardness, and water temperature were 7.5–7.8 mg L^−1^, 7.7–7.9, 130–290 µScm^−1^, 120 mg L^−1^ (as CaCO_3_), and 22–24°C, respectively. Concentrations of Hg, Cu, Cd, and Zn were not detected in the dilution water. All chemical reagents used in the experiment were of analytical grade, and the purity of HgCl_2_, CuSO_4_•5H_2_O, Cd(NO_3_)_2_•4H_2_O, and ZnCl_2_ were 99.50%, 99.0%, 99.0%, and 98.0%, respectively.

### Testing Equipment

A biological early warning system, manufactured by Biological Monitoring, Inc., USA (model: Bio-sensor 7008), was used. The main principles of its use are to use fish as indicator organisms, monitor ventilatory frequency and amplitude of fish by sensors, and to provide a warning when the aquatic environment changes according to changes in the ventilatory parameters. The early warning system consists of 5 parts: (1) ventilatory monitoring sensor (Bio-Sensor), (2) signal filter and amplifier (Bio-Amp), (3) computer data processing and display system, (4) YSI water quality analyzer, and (5) automatic alarm device and water sampler. Operculum respiration and other neuromuscular activities of fish generate several microvolt bioelectrical signals, the strongest of which is a respiratory signal. The signal is received by the sensor in the respiratory chamber and is then sent to signal filter and amplifier (Bio-Amp) before being transferred to a computer. The computer makes judgments as to outliers according to default statistical methods and sends out early warning signals, providing continuous, online monitoring of water pollution[16]–[17]. The system automatically collects water samples after an alarm and determines water quality by chemical analyses.

### Testing Procedures

#### LC_50_ assessment

Before the official experiment, we conducted a preliminary experiment by using a hydrostatic method. To do this, we prepared a wide concentration series of experimental liquids, recorded the quantity of dead fish for each concentration every 6 h, and promptly removed dead fish. Each experiment lasted 96 h, and we selected the minimum lethal concentration for the experimental fish (24 h LC_100_), and the maximum tolerated concentration (96 h LC_0_). The series concentration of the official experiment ranged from 24 h LC_100_ to 96 h LC_0_ with geometric series as interval, set up 5 groups of concentration gradients, and established 3 series and 1 blank control in each group. The experimental liquid volume was 15 L, and there were 10 fish per tank. Fish were not fed during the experiment; poisoning symptoms, time of death time, and number of deaths were recorded. The fish were observed continuously for 96 additional hours, with dead fish and metabolites being promptly removed. The standard to determine death in experimental fish was a lack of reaction upon prodding the fish’s tail with a glass bar [18].

#### Ventilatory responses

The experimental method was according to ASTM [15]. Experimental fish were put into 8 monitoring chambers after 14 days of accommodation, to conduct a respiratory adaptability experiment for 4 days in control water. A standard solution of heavy metals was configured for a series of concentration gradients. The ratio of heavy metal concentration to the corresponding 96 h LC_50_ was defined as U. U-values for each metal were 0, 0.05, 0.1, 0.2, 0.4, 0.8, and 1.6, respectively. The tested standard solution was introduced into the monitoring chambers at a constant flow (100 mLmin^−1^) by using a metering pump, and respiration reaction experiments were conducted for every concentration for 1 h. The signal values of fish respiratory reactions (ventilatory frequency Vf and ventilatory amplitude Va) were recorded for the conditions of control water and heavy metal exposure.

### Data Analysis

We calculated the median lethal concentration (LC_50_) of the acute toxicity experiment and 95% confidence intervals with probability unit regression by using the PROBIT function of SPSS 16.0 software (SPSS Inc., USA). VF and VA data were analyzed with one-way analysis of variance (ANOVA) to determine whether significant differences existed among experimental groups (SPSS 16.0). If a difference was significant, Duncan’s test of multiple comparison was applied. *P* values of <0.05 were considered significant. Early fish biological statistical algorithms using the moving average method were applied to set up an evaluation interval of 8 min with 6 statistical calculation samples [10]. The relative sensitivity of the behavioral responses was evaluated by comparing their threshold values. Threshold effect concentration (TEC) was estimated by defining the geometric mean between the lowest observed effect concentration (LOEC) and the no observed effect concentration (NOEC) [19].

## Results

### Effect of Acute Lethal Toxicity on 3 Cyprinid Fish Species Exposed to Heavy Metals

**Figure 1:**
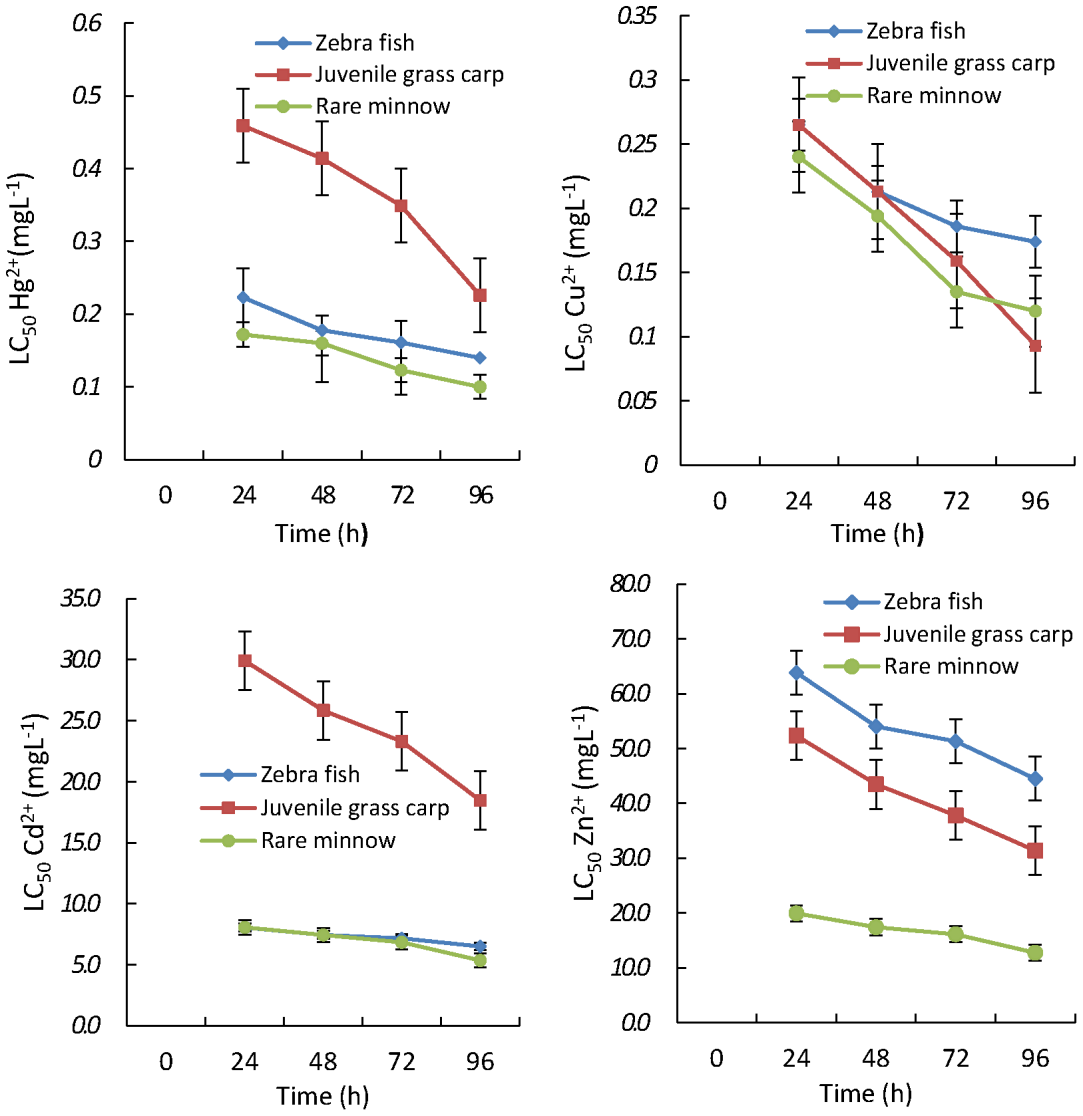
LC_50_ values (mgL^−**1**^) over time for zebrafish (*Brachydanio rerio)*, rare Minnow *(Gobiocypris rarus)* and juvenile grass carp *(Ctenopharyngodon idellus)* exposed to 4 kinds of heavy metals.

During the experiment, no fish in the control group died, and the acute lethal effect caused by the 4 heavy metals on the 3 species of fish are shown in Figure 1. The 96 h LC_50_ (and 95% confidence intervals) values of zebra fish, grass carp, and juvenile rare minnow were 0.14 (0.068–0.268) mg L^−1^, 0.23 (0.171–0.295) mg L^−1^, and 0.10 (0.076–0.131) mg L^−1^, respectively, when exposed to Hg^2+^. The 96 h LC_50_ values of zebra fish, grass carp, juvenile rare minnow were 0.17 (0.102–0.29) mg L^−1^, 0.09 (0.065–0.124) mg L^−1^, and 0.12 (0.075–0.175) mg L^−1^, respectively, when exposed to Cu^2+^. The 96 h LC_50_ values caused by Cd^2+^ were 6.5 (6.15–6.83) mg L^−1^, 18.47 (13.55–25.09) mg L^−1^, and 5.36 (1.58–9.12) mg L^−1^, respectively. The 96 h LC_50_ values caused by Zn^2+^ were 44.48 (36.7–52.7) mg L^−1^, 31.37 (25.74–38.27) mg L^−1^, and 12.74 (4.16–23.9) mg L^−1^, respectively. The fitting equation of acute toxicity showed a good linear relationship among the zebra fish, grass carp, and juvenile rare minnow for the 4 heavy metals, and the related coefficients were 0.90–0.99, 0.88–0.99, and 0.92–0.99, respectively, which showed that the acute lethal effect exhibited a significant dose-effect relationship. Figure 1 indicates that LC_50_ decreased with increasing exposure time and that the toxicity of heavy metals to the fish also demonstrated a significant time-effect relationship.

### The Effect of Heavy Metals on the Respiratory Behaviors of Fish

**Figure 2:**
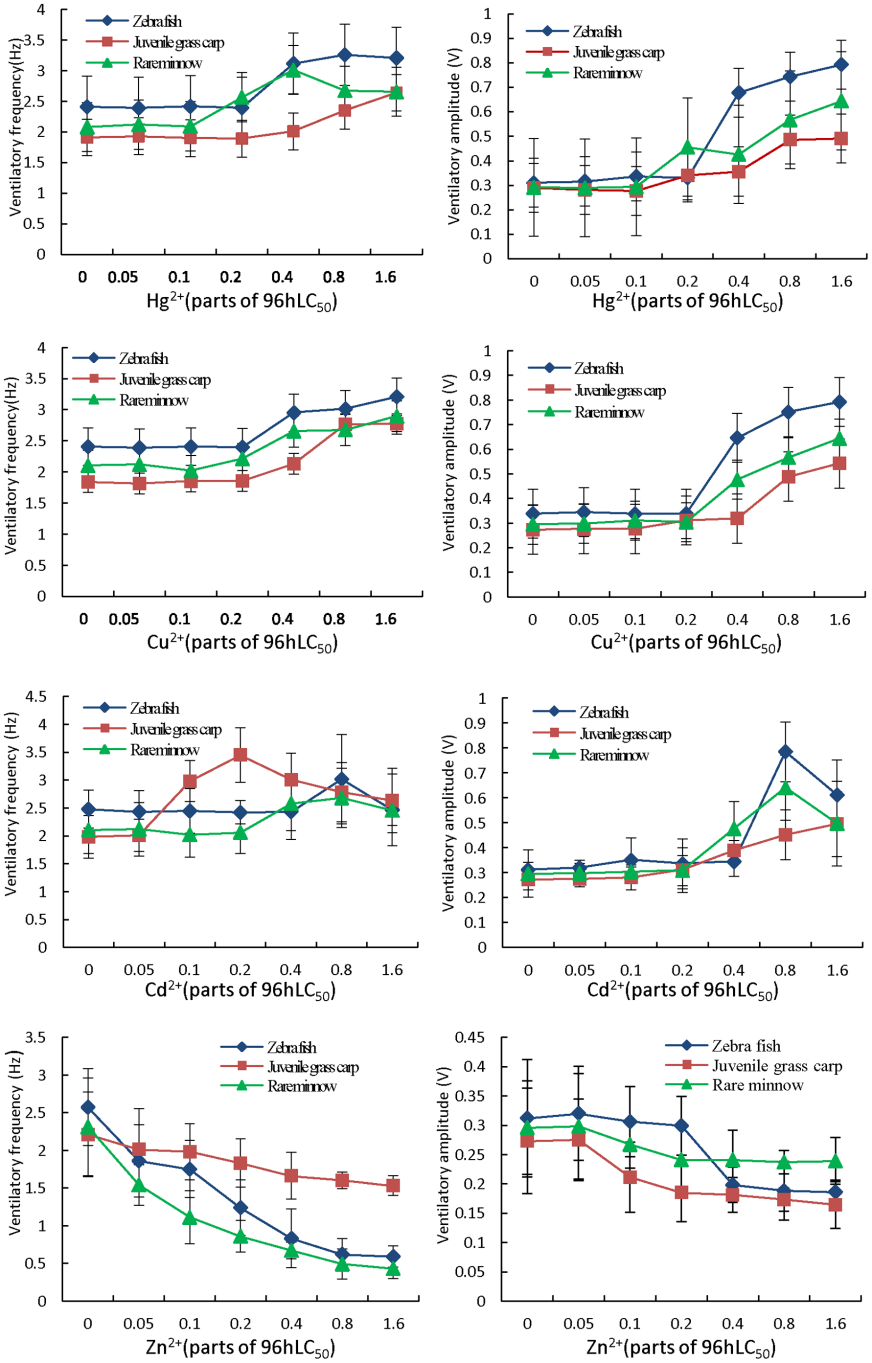
Ventilatory responses over time for zebrafish (*Brachydanio rerio)*, rare minnow *(Gobiocypris rarus)* and juvenile grass carp *(Ctenopharyngodon idellus)* exposed to 4 kinds of heavy metals.

Compared to the control, the mean Vf and Va of the 3 species of cyprinid fish exposed to Hg^2+^ generally increased with increasing concentration (Fig. 2). It can be seen from Figure 2 that with an increase in exposure concentration, Hg^2+^ will stimulate the Vf and Va of each fish species. When the ratios of Hg^2+^ concentration to its 96 h LC_50_ (U) were 0.05, 0.1, and 0.2, the Vf and Va of zebra fish tended to increase, although not significantly. When the U-value increased to 0.4, 0.8, and 1.6, respectively, Vf and Va of zebra fish increased significantly (P<0.05). When the U-value was 0.8 (i.e., the concentration of Hg^2+^ was 0.112 mg L^−1^), Vf reached its highest level, increasing from 2.41 Hz in the control to 3.26 Hz, with 35.2% increase. When the U-value was 1.6 (i.e., the concentration of Hg^2+^ was 0.224 mg/L), Va reached its highest level, increasing from 0.31 V in the control to 0.79 V, with 155.6% increase.

When exposed to Hg^2+^ with an U-value of 0.8, the Vf of juvenile grass carp increased significantly (P<0.01); when the U-value was 0.2, its Va increased significantly (P<0.05). When the U-value exceeded 0.1, Vf and Va of the rare minnow increased significantly (P<0.01) compared to the control group.

When exposed to Cu^2+^, zebra fish, juvenile grass carp, and rare minnow had similar respiratory responses as when exposed to Hg^2+^. When U-values were less than 0.2, the values of Vf and Va showed no significant difference; when the U-value was greater than 0.4, the values of Vf and Va increased significantly (P<0.01).

With the rise of concentration in Cd^2+^ exposure, the values of Vf and Va for each fish species increased initially, followed by a decrease. When the U-value of juvenile grass carp exceeded 0.05, the Vf value increased prominently (P<0.01), reaching a maximum at U = 0.2 and subsequently decreasing. Nevertheless, Vf was still significantly higher than that of the control group (P<0.05). Compared to Vf, Va had a lagged effect; zebra fish and rare minnows showed a similar response, but with different exposure response concentrations.

Compared to the other heavy metals, Zn^2+^ had different toxic effects. The Vf of all fish species decreased with the increase in exposure concentration (P<0.01); the Vf value of rare minnows had an obvious dose-effect relationship with exposure concentration (Y = 2.758 e^−0. 28 x^, R^2^ = 0.98). With the increase in Zn^2+^ exposure concentration, the Va values of all fish were significantly reduced (P<0.05); the Va value of rare minnows declined most dramatically (P<0.05) with U-values greater than 0.05, while it tended to be stable with a U-value of 0.2.

## Discussion

In this study, we analyzed the sensitivity of 4 heavy metals in experimental fish and have shown that the respiratory parameters of rare minnow can provide basic data for monitoring heavy metal pollution in water bodies. Different species of fish have been used to monitor the pollution of water bodies [6], [8], [11] However, These fish species reported in the literature are hard to find or hard to raise in China. In this study, we used two widespread Chinese native species and one international standard species to monitor the water body pollution. We have shown that these species are sensitive to the 4 heavy metals, which are the major pollutants.

Evidence has shown that the toxicity of pollutants is correlated among different aquatic organisms. Firth [20] used different kinds of biology (Rainbow-Trout and Ceriodaphnia) to determine the toxicity of wastewater discharged by a paper mill and found that the toxicity of different kinds of biology had good linear relativity. On the basis of experimental studies, Jiang Min et al. [21] discovered that the influence of heterocyclic nitrogen on the toxicity of luminous bacteria fit that of its effect on zebra fish and daphnia magna, with a correlation coefficient of greater than 0.99. In this study, we selected zebra fish, rare minnow, and grass carp as the standard fish for the experiment. Grass carp is one of 4 Chinese carp species. The rare minnow is a rare cyprinid fish in China that represent small- and medium-sized fish, respectively, from different aquatic habitats. As shown in Table 1, the 4 heavy metals yielded similar acute toxicity values among the 3 fish species (P<0.05), with R values of 0.977, 0.960, and 0.985, respectively. This correlation analysis indicated that the toxic effect of each heavy metal on a given cyprinid fish species was similar and that there is little effect of habitat and size at maturity on heavy metal toxicity. As a result, reliable toxicity data can be obtained on the basis of acute toxicity in early life stages.

**Table 1:** Correlation analysis among the toxicity of the 4 heavy metals to the 3 Cyprinid Fish species.

Species		Zebra fish	Juvenile grass carp	Rare minnow
Zebra fish	Pearson Correlation	1	0.977**	0.960**
	Sig. (2-tailed)		0.000	0.000
	N	20	20	20
Juvenile grass carp	Pearson Correlation	0.977**	1	0.985**
	Sig. (2-tailed)	0.000		0.000
	N	20	20	20
Rare minnow	Pearson Correlation	0.960**	0.985**	1
	Sig. (2-tailed)	0.000	0.000	
	N	20	20	20

** Correlation is significant at the 0.01 level (2-tailed).

The lethal effect of Hg^2+^, which was highest on zebra fish, was higher than that reported for fathead minnow [22]. As for Cu^2+^, its toxicity was highest on juvenile grass carp, and its 96 h LC_50_ was 0.09 mg L^−1^, far lower than 1.1 mg L^−1^ reported for bluegill [23] and also lower than 0.1 mg L^−1^ reported for brook charr [24]. Among the 3 cyprinid fishes, both Cd^2+^ and Zn^2+^ produced the highest mortality rate in the rare minnow. The 96 h LC_50_ of Cd^2+^ for rare minnow was lower than that for bluegill [25] and fathead minnow [26] but higher than that for flag fish [27]. The 96 h LC_50_ of Zn^2+^ for rare minnow was 12.74 mg L^−1^, which was higher than 9.20 mg L^−1^ for fathead minnow [28].

Our study has found that, compared with the acute lethal effect, the heavy metal ion exhibited a greater effect on fish breathing. Gills are the major target organ for water-borne pollutants, and they are the site for metal uptake [5]. As shown in Table 2, of the 3 cyprinid species studied, rare minnow was the most sensitive to Hg^2+^, Cu^2+^, and Zn^2+^, while the TEC of Hg^2+^ on rare minnow was 0.019 mg L^−1^ (i.e., 19% of its 96 h LC_50_), which was close to the sensitivity of the breathing effect on largemouth bass [29]. The response time of the 3 cyprinid fish was between 32 and 56 min. The Cu^2+^ LOEC was the same as that for largemouth bass, but the sensitivity to Zn^2+^ was higher than that of both rainbow trout [30] and brook charr [31]. Therefore, it was very appropriate to select the rare minnow as China’s native experimental fish for the study on respiratory toxicology. Meanwhile, we observed that the ventilation frequency of rare minnow was noticeably related to the dosage of Zn^2+^, and the relationship between dosage and effect was expressed as Y = 2.7588e^−0.28x^, R^2^ = 0.986. Gintaras Svecevicius [17] exposed rainbow trout to hexavalent chromium and found a noticeable relationship between dosage and effect, i.e., Y = 98.388/(1+0.152e^6.9844x^), R^2^ = 0.98, P<0.05). In addition, the TEC was 0.2 mg CrL^−1^, only 7% of the 96 h LC_50_ coefficient, leading Svecevicius to believe that ventilation frequency could be used as a biomarker in the standard test of the toxicity of water.

**Table 2:** Analysis of the acute toxicity, respiratory reaction and sensitivity of different fish species.

	Species	NOEC	LOEC	TEC	96 hLC_50_	Parts of 96 hLC_50_	Hardness(mg/L CaCO_3_)	pH	Reference
Hg^2+^	zebra fish	0.05	0.056	0.053	0.14	0.38	120	7.8	This paper
	rare Minnow	0.018	0.02	0.019	0.1	0.19	120	7.8	This paper
	grass carp	0.041	0.046	0.043	0.23	0.19	120	7.8	This paper
	largemouth bass		0.01					7	[29]
	fathead minnow				0.168		45	7.4	[22]
Cu^2+^	zebra fish	0.062	0.068	0.065	0.17	0.38	120	7.8	This paper
	rare Minnow	0.045	0.048	0.046	0.12	0.39	120	7.8	This paper
	grass carp	0.066	0.072	0.069	0.09	0.77	120	7.8	This paper
	largemouth bass		0.048					7	[29]
	Bluegill				1.1		45	7.5	[23]
	brook charr				0.1		45	7.5	[24]
Cd^2+^	zebra fish	2.5	2.6	2.55	6.5	0.39	120	7.8	This paper
	rare Minnow	2.14	2.144	2.142	5.36	0.4	120	7.8	This paper
	grass carp	1.84	1.847	1.843	18.47	0.1	120	7.8	This paper
	rainbow trout		0.064				82	7.8	[32]
	largemouth bass		0.15					7	[29]
	Bluegill				20.4		200	7.7	[25]
	fathead minnow				7.2		200	7.7	[26]
	flag fish				2.5		44	7.5	[27]
Zn^2+^	zebra fish	2.2	2.224	2.212	44.48	0.05	120	7.8	This paper
	rare Minnow	0.629	0.637	0.633	12.74	0.05	120	7.8	This paper
	grass carp	3.12	3.137	3.128	31.37	0.1	120	7.8	This paper
	fathead minnow				9.20		203	7.7	[28]
	rainbow trout		2.55				50	7.8	[30]
	Brook charr		1.39				45	7.5	[31]

Moreover, the 4 heavy metal ions stimulated different types of respiratory reactions in the experimental fish. Hg^2+^ and Cu^2+^ were both noticeably stimulative on Vf and Va of each of the fish species; when fish were exposed to an increased concentration of ions. Both Vf and Va of Cd^2+^ initially rose and then gradually dropped. Zn^2+^ had an obviously different toxicity effect from that of the other heavy metal ions, as both its Vf and Va dropped gradually, which is similar to the conclusion drawn by Diamond et al. for bluegill [32]. Different types of heavy metal ions have different effects on fish respiration. The mechanism by which these toxic substances exert their influence on fish respiration is still unclear and needs to be explored.

### Conclusions

The acute toxicities of the 4 heavy metal ions on zebra fish, juvenile grass carp, and rare minnow were linearly correlated, indicating clear relationships between dosage and effect as well as between time and effect. The respiratory activity of the fish was highly sensitive to heavy metal pollution, and this sensitivity was much greater than the lethal reaction. Among the 3 cyprinid species studied, rare minnow was most sensitive to heavy metals. It is feasible to use the respiratory parameter of the rare minnow as a biomarker for evaluating the toxicity of heavy metals and to utilize this species as a sensor to monitor and predict heavy metal pollution.
